# Combined empowerment, continuous appraisal, psychological and career counseling improve medical cadets' satisfaction and may potentially offset burnout during internship year. An 18-months successful experience in a tertiary medical center

**DOI:** 10.3389/fpubh.2024.1432571

**Published:** 2024-11-27

**Authors:** Reut Shoham, Hanni Robinson, Noy Yehiel, Vered Robinzon, Yael Frenkel Nir, Gad Segal

**Affiliations:** ^1^Education Authority, Chaim Sheba Medical Center, Affiliated to the Faculty of Medicine, Tel-Aviv University, Tel-Aviv, Israel; ^2^Faculty of Medicine at the Hebrew University, Jerusalem, Israel; ^3^Management Wing, Chaim Sheba Medical Center, Affiliated to the Faculty of Medicine, Tel-Aviv University, Tel-Aviv, Israel

**Keywords:** interns, burnout, empowerment, satisfaction, appraisal, career counseling

## Abstract

**Background:**

Following graduation, Israeli medical cadets complete a 12-months training period in hospitals, having profound influence on their future career. Burnout, sense of personal and professional uncertainty and disorientation are already notable in this group. The aim of the current study was to assess the potential impact of empowerment, psychological and career counseling on the level of satisfaction and burnout of medicine cadets during their internship year in a tertiary medical center.

**Methods:**

In a tertiary hospital's education authority, we offer constructed, personalized psychological and counseling services with continuous process of appraisal. During an 18-month period we followed their monthly feedback relating to satisfaction, learning experience and level of socialization. The study was designed as comparative research assessing cadets' satisfaction before, and after intervention as a surrogate marker for their burnout.

**Results:**

Comparison of measured parameters showed statistically significant improvement, with interns stationed in the surgical departments (*n* = 86) showing the highest degree of improvement: the extent of acquiring new knowledge and competencies (1 to 5 Likert) went from 2.2 ± 1.0 to 3.3 ± 1.42; *p* < 0.005; experience from the absorption process into the department (LQR from 2.6 ± 1.2 to 3.5 ± 1.56; *p* < 0.05), degree of motivation to recommend peers to apply for residency (LQR from 2.3 ± 1.0 to 3.1 ± 1.6; *p* = 0.05) and the experience of being accepted to the department by the staff nurses (LQR from 2.7 ± 1.3 to 3.5 ± 1.1; *p* < 0.05).

**Conclusions:**

A combined, ongoing process of appraisal, empowerment, psychological and career counseling seems promising in the relenting effort to improve cadets' satisfaction and hopefully withhold the burnout process of young physicians.

## Introduction

The main problem addressed by this research was the low satisfaction and accelerated burnout amongst medicine cadets during their internship year. We strived to establish effective means for counterbalancing these psychological pathologies amongst this precious population of young physicians.

### Characteristics of young physician cadets at their year of internship

The internship period during the pathway of medical education is an essential element that enhances skills and knowledge in clinical practice. In Israel, 1-year intership program is a pivotal part, signing the transformation of graduates from students to certified physicians. During this period of 12-month training, the young medicine-school graduates are expected to both learn and work as part of the hospitals' team of physicians, rotating between different, compulsory, and elective departments. Compulsory 9 months are divided to training in departments of internal medicine (3 months), emergency medicine department (1 month), department of general surgery (2 month), anesthesiology and intensive unit care (1 month), and 2 months in pediatrics. The remaining 2 months are electives, primarily used by interns to get better familiarity with specific departments, in terms of both the clinical discipline and the personnel. Additionally, 1 month is appointed for vacation, completing the full 12-months duration. This 1-year training program aims to expand the interns' knowledge of the cornerstones of medicine while exposing them to various clinical practice settings. This exposure helps in their decision of future residency and career paths, offering the opportunity to practice medicine in a supervised environment (1). This is a true year of duality: young physicians are expected to both learn and work at the same time and these contradictory vectors put them in substantial physiological and psychological stress.

### The burnout of medical professionals begin early in career

The process of staff burnout seems to be the most endangering for the future of healthcare systems both locally in Israel, as depicted by the national program executed by the Israeli Ministry of Health in the year 2021 (2) and worldwide: it affects more than half of these systems' workforce and is associated with negative outcomes for both patients and health-systems (3). Young physicians are especially affected with depression and dissatisfaction being the phenotypes of burnout in this population (4, 5). Gunasingam et al. associated young physicians' burnout with senses of dissatisfaction and suggested potential benefits of debriefing meetings (6). There was a surge in the research of healthcare professionals' burnout during, and as a result of the COVID-19 pandemic with young physicians displaying rapidly progressive burnout. Such publications also focused attention on the need to implement sustainable wellbeing improvements in young physicians' working conditions (7, 8). The association between physicians' burnout and sense of wellbeing described may enable the appreciation of burnout by measurements of wellbeing, as subjectively reported by students, interns and physicians (9).

### The potential role of carrier counseling for medical cadets during their internship year

In their thorough review, Scott et al. (10) note that career decisions taken by pre-graduate, medical students are inclined to external influences. Notwithstanding, there are scarce evidence that indeed, such external influences, taking the format of mentoring programs succeed in helping this population. Scott et al. did not deter from the above and still recommended investing time and efforts in designated career advising programs when the students are nearing graduation (10). Another rational for career counseling at an early stage is supported by the findings of Michael J. Goldacre et al. In their long-observed study, they followed the early career choices of medical graduates in the United Kingdom. They found that 10 years after qualification about a quarter of doctors were working in a specialty that was different from the one chosen in their third year after graduation. In their perspective this situation requires both flexibility on one hand, and creating the ability to help graduates to progress quickly into their chosen specialty once they are definite with their choice. This shows that the road young graduates go through during their time in the medical education system can have profound effects on their career choices. Our efforts, therefore, engaging with career counseling during internship year, stand in line with the professional literature (11).

### The potential role of ongoing appraisal of interns' perspectives

Mataya et al. shared their experience in a longitudinal effort of following the interns' perspectives and level of satisfaction, based on consecutive questionnaires (12), standing for passive data mining regarding satisfaction. Complementing this passive data mining, and standing for active interventions, Lachish and co. found out, that continuous institutional support has a significant positive effect on safeguarding and long-term retention of junior physicians (13, 14). Several earlier publications highlight the importance of questionnaires in understanding the experiences and views of physicians during training, which may ultimately make improvements in their working conditions and overall wellbeing (15–17). Integration of the above perspectives shed light on the need for continuous interns' satisfaction measurement using questionnaires, accompanied by supporting the cadets by continuous positive appraisal.

### Empowering medical cadets with clinical responsibilities during their internship year

In the qualitative study conducted by Sophie Querido and Co., Experiencing clinical responsibility was found to be a key factor for career preference (18). Their conclusion was that by receiving clinical responsibility the students are forced to reflect on their personal needs and to think in depth about the best career choice for them. One of the goals of the 1-year internship in Israel is indeed, to help interns achieve better career decisions by exposing them to different clinical disciplines. Nevertheless, empowerment of interns is not always the rule in all departments. This should be actively looked for and implemented by the physicians responsible for interns during their daily life routines.

In light of all of the above, we decided to peruse an ongoing appraisal of our hospitals' cadets during an 18-month period with three main interventions:

a) Early in career psychological and job counseling all through the internship year.
b) Ongoing empowerment of interns during their work in the hospital departments.
c) Ongoing appraisal and follow-up of interns' perspectives.

The aim of the current study was to assess the potential impact of empowerment, psychological and career counseling on the level of satisfaction and burnout of medicine cadets during their internship year in a tertiary medical center. We hypothesized that such interventions, based on empowerment and counseling would increase our young physicians' satisfaction and assimilation in their clinical placements. Since we decided to implement our intervention to the whole cadets' population in our medical center, we compared satisfaction parameters in the entire population, before and after interventions. Eventually, we strive at halting and ending the process of their personal and professional burnout, with satisfaction serving as a surrogate marker for this important end-point.

## Methods

### Data collection and analysis

The study was approved by an institutional review board (IRB, approval number (SMC 0770-23). This study was designed as comparative research assessing cadets' satisfaction measurements before, and after intervention as a surrogate marker for their burnout. This study design, of internal-group comparison, pre- and post-intervention, was supposed to minimize concerns about confounders and optional explanations to the comparison results. Data was prospectively collected using the following inclusion criteria: Interns had to answer an online internet survey, which was sent by the end of each calendric month via a social network group of all interns in the Chaim Sheba Medical Hospital (150–200 interns at any point in time) stationed in the following hospital departments: internal medicine, emergency room, general surgery, pediatric including pediatric intensive care, elective departments, anesthesia and intensive care unit. Since internes are rotating between the above departments every 1–2 months, we assumed that tenure did not affect their responses along their internship year. The survey was anonymous, enabling each intern to answer only once monthly. The questionnaire had two demographical questions to help decide whether such factors may be associated with certain answers: (a). place of medical school graduation (either in Israel or abroad), (b). gender (male of female). The following six quantitative questions (answered on a Likert scale) were: (a) rate the experience from the process of your absorption into the department, (b) rate the experience from the manner of your being accepted to the department by staff physicians, (c) rate the experience from the manner of your being accepted to the department by staff nurses, (d) rate the extent to which you got new knowledge and competencies during your stay in the department, (e) rate your degree of motivation to ask for residency in the department, and (f) rate the degree of your recommendation to peers to become interns in this department. Two qualitative, open questions were also asked: (a) specify aspects of your experience in the department that were positive and therefore, should be preserved, and (b) specify aspects of your experience in the department that were negative and therefore, should be improved.

### Applied interventions

#### Early in career psychological and job counseling all through the internship year

Each intern had three personal meetings along his 12-month stay in our medical center. The first one is dedicated for personal acquaintance, and for devising his/her personal program of internship during the following year (deciding on the stepwise manner of his/her progression through the different hospital departments and choosing the placements for two elective months). The two following meetings are dedicated for follow-up and management of accumulated experiences (positive and negative) during the intern's stay in different hospital departments. Relevant competencies are discussed in order to endow the intern with practical tools for better self-management in his/her unfamiliar environment. Also, thoughts and lessons learned are used in order to better the intern's intentions for post-internship residency. Decisions taken prior to internship are often reconsidered. All meetings are done by a specialized, organizational psychologist, keeping close correlation with the administrators responsible for the interns about their daily routines and well-acquainted with the full spectrum of career options for Israeli physicians.

#### Ongoing empowerment of interns during their work in the hospital departments

In each and every department, a senior resident or young specialist was assigned as responsible for the interns in that specific department. As a group, these “agents” are being watched, gathered, and guided so they gain competencies and tools needed for interns' empowerment. These physicians are also meeting the psychologist whenever a question arises about the professional and personal competence of interns. Also, interns are widely involved in teaching projects in our medical center. They are guided and taught competencies for becoming CCA (Clinical Competency Assessment) [previously known as OSCE (objective structured clinical evaluation)] examiners and are also encouraged to take part in many pre-graduate tutorial missions. Also, interns are invited to take part in courses that empower their competencies, including a GCP (good clinical practice) course that enable them to take part in clinical studies as an official investigator and also as principal investigators whenever appropriate. The interns are going through workshops that are preparing them for better performance in interviews, which are a critical element in residency application.

#### Ongoing appraisal and follow-up of interns' perspectives

All along their 12-months stay in our medical center, an ongoing process of follow-up and appraisal is taking place: either during the activities mentioned above and by using a monthly questionnaire that is sent by email and WhatsApp application. Therefore, the interns experience our continuous follow-up, appraisal and frequent feedback. Interns feedback was summarized and points for either improvement or preservation were communicated to the either the resident or senior physician, in each department, that was in charge of the interns at that point in time.

### Statistical analysis

All questionnaires answered were anonymized and coded and afterwards, numeric data were extracted. Non-parametric Kruskal-Wallis test was used for multiple groups and the Tukey-Kramer comparison was added to allow the source of variance determination. Also, the non-parametric Wilcoxon-Mann-Whitney test was used for comparison of ordinal variables splitting into two groups, as well as *T*-test were used. A significant difference was defined as a *p*-value of < 0.05 and led to the rejection of the null hypothesis in favor of the alternative hypothesis. The confidence interval was defined at the 95% level. Date analysis for the six quantitative questionnaire items was performed using the software programs: Microsoft Excel and Analyze-it for Microsoft Excel 5.9, 2023, The Tannery, 91 Kirkstall Road, Leeds, LS3 1HS, United Kingdom.

## Results

During the study period, May 2022 and October 2023, a total of 423 questionnaires were sent. Each intern could answer once in any certain point of time but there was no barrier for the same intern to answer in consecutive months. Therefore, we do not know the total number of interns that took part in this study. Quantitate questions' results were plotted along the timeline of 18-months and a trend line was calculated along the study period. We calculated the statistical significance of trends along the study.

### Quantitative questionnaires' analysis

**Figure 1:**
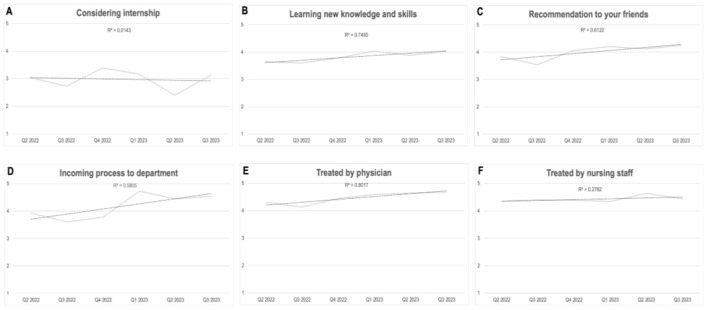
**(A–F)** 18-months changes in interns feedback. Internal medicine departments.

**Figure 2:**
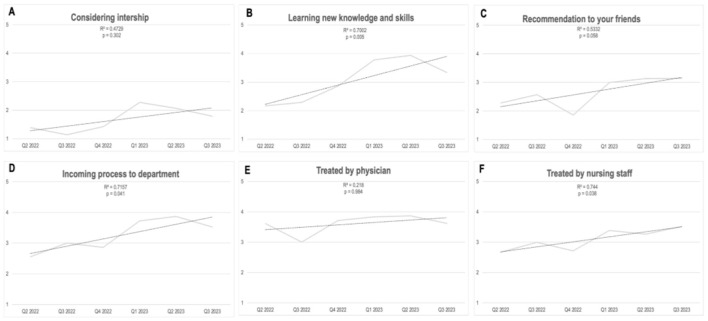
**(A–F)** 18-months changes in interns feedback. Surgery wing departments.

Analysis of the interns' answers to their monthly questionnaires was done on a monthly basis and along the temporal line of 18 months' duration. Analysis of the results show that during their stay in internal medicine departments positive feedback was generally maintained (Figure 1), feedback from the surgical wing departments showed a sustainable, statistically significant improvement along the study period (Figure 2).

Statistical significance with an improvement of intern's satisfaction was found in several Likert (LQR) questions in the surgery wing departments: experience from the absorption process into the department (LQR went from 2.6 ± 1.2 to 3.5 ± 1.56; *p* < 0.05), the extent of acquiring new knowledge and competencies (LQR went from 2.2 ± 1.0 to 3.3 ± 1.42; *p* < 0.01), degree of motivation to recommend peers to apply for residency (LQR from 2.3 ± 1.0 to 3.1 ± 1.6; *p* = 0.05) and the experience of being accepted to the department by the staff nurses (LQR from 2.7 ± 1.3 to 3.5 ± 1.1; *p* < 0.05).

In the internal medicine departments, statistical significance was found for the trend of improvement in the interns' assessment of the experience from the absorption process into the department: there was an improvement along the year (Figure 2D; *p* < 0.01).

With regards to other hospital departments, we saw that whenever the stay in a certain department was short (1 month in the emergency department and in the anesthesia department) there was no improvement during the study duration. Also, in the pediatric departments, where the satisfaction of interns was high along the whole study period, there was also, naturally, no significant improvement.

### Multiple variance analysis

Our population of interns is heterogenous (relating to gender and place of medicine school graduation) and therefore, we wanted to assess the possible associations between such interns' demographics and our survey results. We found out that relating to the extent of acquiring new knowledge and competencies, females who graduated medical school in a foreign country gave higher scores throughout their stay in all hospital departments compared to females who graduated from local medical schools (mean difference of 0.8 points; 95% CI; 0.3–1.2; *p* < 0.001). Also, we found out that both females and males who graduated abroad were more satisfied (in their total satisfaction scores) compared to those who graduated locally: for comparison of female graduate (*p* < 0.01) and male graduates (*p* < 0.01).

### Open, qualitative feedback

The survey included two open-ended queries that asked interns to highlight the strengths worth keeping and areas for improvement within their departments. The interns valued factors like teaching, mentoring, atmosphere, and firsthand experience. The overall rate of answering these questions did not allow a thorough analysis and therefore, was omitted from the results chapter in this manuscript.

## Discussion

By the year 2030, there will be a projected global shortage of 18 million healthcare workers (19). In Israel particularly, reduction in the availability of healthcare personnel is expected over the coming years, especially physicians and in certain specialized domains, i.e., anesthesia (20) as well as in the state periphery (21–23). To address this expected deficit, several strategic measures may be implemented: It was repeatedly suggested to offer financial and other incentives to attract physicians to areas of need, re-hiring retired physicians that would return to work, but sufficient solutions are still missing.

Beyond the mere deficit in numbers, there are major concerns of the escalated burnout process of healthcare workers in general and of physicians in particular (5). The COVID-19 pandemic has augmented these challenges to an unprecedented level across the globe (24). This crisis has placed an overwhelming burden on healthcare facilities. Health care professionals faced intense pressure, resulting in significant mental health impacts and burnout due to heavy workloads, limited resources, and increased exposure risks. This eventually caused many healthcare professionals to retire early or consider leaving their professions (24). This highlights the critical need for workforce planning, better support mechanisms for healthcare professionals, and a better understanding of the extensive impacts of the COVID-19 pandemic on healthcare systems, especially in relation to burnout and future workforce shortages among senior physicians and residents.

Upon addressing healthcare professionals' burnout, the means for quantifying the process should be addressed. Several earlier publications associated burnout with the sense of dissatisfaction amongst physicians (6, 9). Therefore, in the current study we decided to measure and quantify interns' satisfaction as a surrogate marker for their burnout process. We addressed these challenges with action plans accompanied by research. Our decision was based on earlier publications stressing the need for organizational interventions aimed at struggling burnout and stress among trainee physicians (25). The results presented in this article show that addressing three essential components in the young physicians' experiences are beneficial: endowing continuous, personal consultation along with professional empowerment and a continuous process of appraisal. Our 18-months' follow-up results did not come to us by surprise: we knew that the biggest challenge would be found in the surgery departments and indeed, the improvement was the most significant in these interns' placements.

Surgery departments are the most challenging to young medicine-school graduates since they face them with their largest gap in competencies, therefore, the level of empowerment and sense of competence are exceptionally low to begin with. Also, the more practical/executive nature of these departments is significantly more stressful for young physicians. Rozenberg et al. (26) suggested, as part of what they named as “Surgical intern wellness initiative”, the usage of a twice monthly reflective questionnaire. This is somewhat equivalent to our ongoing appraisal plus continuous consultation meeting in the current study.

Other, non-surgical departments had relatively high scores to begin with (such as internal medicine and pediatrics). High initial scores for theses departments created a ceiling effect in the Likert-type questionnaire that we used. Considering the ceiling effect, one must differentiate between departments that have high initial scores and did not improve much and departments that had low initial scores and did not show major improvement (such as the emergency medicine department). Such departments with low initial scores and small improvements might be suitable for development of other interventions in the future.

Based on the responses provided to the two open-ended queries in this study, it is worth noting that “long working hours” significantly contribute to interns' low levels of satisfaction, undoubtfully accelerating burnout. This negative factor was clear at all hospitals' departments and is in alignment with previous reports and organizational knowledge (27).

## Conclusions

As part of taking care of young physicians during their internship year regarding their bureaucracy matters and organizational duties, hospital managements should invest significant attention to three vectors of care for their interns: continuous career and personal/psychological counseling, empowerment of interns aimed at their acquiring relevant, clinical competencies and continuous appraisal of interns along their 12-months stay in hospital. Such efforts should be directed at the surgical departments in which the level of satisfaction for interns is generally low and accelerated, resultant burnout is expected. Investing such efforts in this young but important part of the healthcare system may, eventually, offset the burnout process of physicians.

## Limitations

We did not enforce our interns to take part in the study and therefore, there could be a selection bias to those interns that were less satisfied and more inclined to participate in the study. The fact that the interns' population was heterogenous relating their stage in internship should dismiss the concern regarding the influence of the year progression on their satisfaction. The study was designed as comparative research assessing cadets' satisfaction measurements before, and after intervention as a surrogate marker for their burnout. We did not involve an external comparison group since the intervention was offered to all our medical center's cadets. Every intern could fill out the questionnaire during his/her internship several times in a monthly manner. Therefore, we do not know the exact number of cadets answering the survey but rather count the number of questionnaires answered.

## Data Availability

The raw data supporting the conclusions of this article will be made available by the authors, without undue reservation.
